# Serum FGF21 levels are associated with brown adipose tissue activity in humans

**DOI:** 10.1038/srep10275

**Published:** 2015-05-18

**Authors:** Mark J.W. Hanssen, Evie Broeders, Ricardo J. Samms, Maarten J. Vosselman, Anouk A.J.J. van der Lans, Christine C. Cheng, Andrew C. Adams, Wouter D. van Marken Lichtenbelt, Patrick Schrauwen

**Affiliations:** 1 Department of Human Biology, NUTRIM School for Nutrition and Translational Research in Metabolism,Maastricht University Medical Centre+(MUMC+), Maastricht, the Netherlands; 2 Department of surgery, Maastricht University Medical Centre+(MUMC+), Maastricht, the Netherlands; 3 Lilly Research Laboratories, Lilly Corporate Center, Indianapolis, IN, USA 46285

## Abstract

The obesity pandemic has spurred a need for novel therapies to prevent and treat metabolic complications. The recent rediscovery of brown adipose tissue (BAT) in humans made this tissue a possible therapeutic target, due to its potentially substantial contributions to energy homeostasis. Fibroblast growth factor 21 (FGF21) has been identified as a facilitator of cold-induced thermogenesis in humans. Furthermore, pre-clinical studies revealed that FGF21 administration leads to improvement in the metabolic consequences of obesity, such as dyslipidemia and type 2 diabetes. Here we studied plasma FGF21 levels in two cohorts of human subjects, in whom BAT activity was determined using an individualized cooling protocol by [^18^F]FDG-PET/CT scan. Importantly, we found that circulating FGF21 levels correlated with BAT activity during acute cold exposure in male subjects. In addition, FGF21 levels were related to the change in core temperature upon acute cold exposure, indicating a role for FGF21 in maintaining normothermia, possibly via activation of BAT. Furthermore, cold acclimation increased BAT activity in parallel with increased FGF21 levels. In conclusion, our results demonstrate that FGF21 levels in humans are related to BAT activity, suggesting that FGF21 may represent a novel mechanism via which BAT activity in humans may be enhanced.

The obesity pandemic is associated with major metabolic disturbances, including type 2 diabetes, non-alcoholic fatty liver disease (NAFLD) and cardiovascular disease. Lifestyle interventions aimed at increasing physical activity and decreasing food intake can be effective in treating these disorders. However, due to poor adherence such interventions are not easily translated to the general population. As such, there is an ongoing need for novel therapies that may alleviate obesity-induced metabolic complications, especially as currently available therapies offer only modest efficacy. Fibroblast growth factor-21 (FGF21) was originally discovered in 2000 as a new member of the FGF superfamily^1^. The first hint at its metabolic effects came about when FGF21 was identified as a factor able to augment glucose uptake in an adipocyte screen^2^. Subsequently, FGF21 was shown to have favorable metabolic effects *in vivo*^3^,^4^. Transgenic mice with FGF21 overexpression in liver demonstrated improved insulin sensitivity and resistance to weight gain on a high-fat diet^2^. Systemic administration of recombinant human FGF21 in mice leads to lowered blood glucose levels, improved insulin-sensitivity^2^, amelioration of dyslipidemia, and preservation of β-cell function^5^. Mice infused with FGF21 also exhibit elevated energy expenditure (EE)^6^,^7^, likely due to a thermogenic response in white (WAT) and brown adipose tissue (BAT)^8^,^9^. Recently, the results of the first phase 1B study using an FGF21 analog, termed LY2405319, conducted in obese type 2 diabetic patients, became available. This trial demonstrated that the majority of FGF21’s metabolic effects are translatable to humans^10^. Specifically, chronic treatment with varying doses of LY2405319 resulted in improvements in plasma lipid profiles, and decreases in fasting insulin levels and body weight.

Since the rediscovery of brown adipose tissue (BAT) in humans in 2009, the search for novel ways to activate BAT in humans has intensified. We^11^ and others^12^ have shown that acute cold is able to promote activation of BAT in the majority of human volunteers. Moreover, cold acclimation for 10 days led to an increased capacity to activate BAT upon acute cold stimulation^13^. The mechanism via which cold activates BAT is not known, nor are all the pathways involved in BAT activation in humans. Alongside more classical activators of BAT, such as thyroid hormone and the sympathetic nervous system (SNS), novel pathways including bone morphogenic proteins (BMPs)^14^,^15^, irisin^16^,^17^ and FGF21^2^,^18^ have been proposed as stimulators of BAT activity and recruitment. Interestingly, cold exposure has been reported to increase circulating levels of FGF21 besides enhanced FGF21 expression in BAT, leading to uncoupling protein-1 (UCP-1) transcription^8^,^19^,^20^.

In addition to activity directly in BAT, FGF21 has recently been demonstrated to stimulate the browning of WAT in a variety of animal studies 2,21,22. Following a three-day cold-exposure challenge, a threefold increase in FGF21 mRNA was observed in BAT in mice^8^, accompanied by a robust induction of FGF21 mRNA in peripheral WAT depots. Furthermore, in human primary beige (or brite) adipocytes FGF21 has been reported to increase thermogenic gene expression and capacity, as indicated by increased basal and uncoupled respiration^23^.

In human subjects, changes in serum FGF21 concentrations correlated positively with cold-induced non-shivering induced thermogenesis^18^. However, so far the relationship between FGF21 and BAT activation has not been studied in vivo in humans. The present study was aimed at elucidating whether elevation of circulating FGF21 levels in humans is associated with the activation of BAT and thereby test if this activation may be in part responsible for the beneficial metabolic effects of FGF21 treatment.

Here, we studied the relationship between circulating FGF21 levels and BAT activity and the effect of acute and prolonged cold exposure (cold acclimation) on these parameters. To this end, we measured plasma FGF21 levels in a large number of subjects from previous studies^13^,^24^,^25^,^26^, who were exposed to an individualized cooling protocol^27^ for evaluation of BAT activity using an [^18^F]FDG-PET/CT scan. Our results reveal that FGF21 levels are positively correlated with BAT activity during acute cold exposure. In addition, chronic cold acclimation increases BAT activity in parallel with an increase in FGF21 levels.

## Methods

### Study protocol

In this study data from 59 lean, healthy subjects, who participated in previously published studies, was used^13^,^24^,^25^,^26^. Written informed consent was obtained from all subjects.

All females were on specific one-phase oral contraceptives (ethinylestradiol/levonorgestrel 20 μg/100 μg) and were not measured during the menstruation period. All subjects underwent an individualized cooling protocol and [^18^F]FDG-PET/CT imaging at Maastricht University Medical Centre for the measurement of BAT activity in the period from April 2010 to June 2013. A total of 25 subjects were exposed to an air-cooling protocol 24,25, while the other 34 subjects underwent a cooling protocol with a water-perfused suit^13^,^26^. Of the latter group, 17 subjects were exposed to an environmental temperature of 15–16 °C for 10 consecutive days: 2 hours on the first day, 4 hours on the second day, and 6 hours per day for the remaining days. BAT activity and FGF21 levels were determined before and after cold acclimatization.

For both the water-cooling protocol and the air-cooling protocol subjects were asked to refrain from heavy exercise 48 hours preceding the test day. In addition, subjects swallowed a telemetric pill (CoreTemp HT150002; HQ Inc) for measurement of core temperature during both protocols.

### Individualized cooling protocols

In the water-cooling protocol, subjects were measured in the early afternoon (starting at 11.45 a.m.) after a 4-hour fasting period. An intravenous cannula was inserted in the antecubital vein, through which blood was sampled in thermoneutral (t = 45 min) and mild cold (t = 120-150 min) conditions, and through which the tracer was administered . After thermoneutral measurements, subjects were exposed to mild cold according to an individualized cooling protocol as described earlier^13^. Briefly, subjects were wrapped in a water-perfused suit. The first 45 minutes subjects were kept at thermoneutral conditions, after which a gradual step-wise decrease in temperature followed (water temperature was lowered with 4 °C every 15 minutes) until shivering occurred. After the onset of shivering, subjects were warmed up for 5 minutes after which the temperature was set slightly above shivering level. Subjects were measured for another 30 minutes during this mild cold exposure. Indirect calorimetry (EZCAL, IDEE, Maastricht University) was used to measure energy expenditure continuously during the cooling protocol. Subsequently, subjects were injected with 75 MBq of [^18^F]FDG and transported to the scanner for [^18^F]FDG-PET/CT imaging for quantification of [^18^F]FDG uptake into BAT. Mild cold exposure was continued while subjects were inside the PET/CT scanner (Gemini TF PET-CT, Philips, The Netherlands).

The 25 subjects that underwent the air-cooling protocol arrived at the laboratory at 08.30 AM after an overnight fast and were placed inside an air permeable climate tent (Colorade Altitude Training, Louisville, CO). Inside this tent the air temperature was regulated by an air conditioner to maintain the air temperature with an accuracy of 1 °C^24^. To guarantee a comfortable position during the measurements, subjects were placed in a semi-supine position. Subjects underwent a personalized cooling protocol^27^ to guarantee maximum non-shivering thermogenesis. Indirect calorimetry was used to measure energy expenditure during three hours. During the first hour (t = 0-60), measurements were performed in thermoneutrality (24-25 °C with standardized clothing, 0.49 clo) followed by a gradual step-wise decrease of the room temperature during the second hour. This was continued until the subjects subjectively reported shivering. At the first signs of shivering, the air temperature was increased by steps of 1 °C until shivering stopped and temperatures were then kept stable at these points. Blood was drawn from the antecubital vein catheter at t = 55 min (during thermoneutrality) and at t = 115 min (during cold-exposure). After one hour of cold exposure (t = 120 min) 50–75 MBq of [^18^F]FDG was injected through an intravenous catheter and cold exposure was continued for another hour, during which subjects were instructed to lay still to prevent uptake of FDG in the muscles. At t = 180 min subjects were transported to the PET/CT-scanner for quantification of [^18^F]FDG uptake into BAT.

### PET/CT scanning protocol

The scanning protocol and data interpretation methods were identical to those used in earlier studies by our group^11^. For the static PET scans (performed in all subjects) subjects were intravenously injected with 50 – 75 MBq (1.35 mCi) of [^18^F]FDG. Sixty minutes after tracer injection imaging started with a low-dose CT scan (120 kV, 30 mAs), immediately followed by a PET scan. A total of six to seven bed positions (5 min per bed position) were necessary to cover the area where BAT is usually found (i.e., abdominal, thoracic, and neck region). The PET image was used to determine the [^18^F]FDG uptake, and the CT image was used for PET attenuation correction and localization of the [^18^F]FDG uptake sites. The voxel size of both reconstructed image sets was 4 × 4 × 4 mm^3^. For the dynamic PET scans (performed only during the water-cooling protocol) subjects were first transported to the PET/CT scanner after which imaging started with a low dose CT scan (30 mAs, 120 kV). Subsequently, subjects were injected with 74 MBq of [^18^F]FDG at the start of the 60-min dynamic PET scanning protocol. Images were reconstructed according to the following time frames: 10 × 15 seconds, 5 × 30 seconds, 5 × 60 seconds, 5 × 120 seconds, and 8 × 300 seconds. After the dynamic PET scan, a static PET scan was performed as described above.

### PET analysis

The scans were analyzed using PMOD software (version 3.0; PMOD Technologies). Both the researcher and an experienced nuclear medicine physician (B.B) interpreted the PET/CT images. Regions of interest were manually outlined, and a threshold of 1.5 SUV (standardized uptake value) and Hounsfield units between –10 and –180 were used to define BAT, as described earlier by our group^13^. For the static PET scans, BAT activity was expressed in standard uptake values (SUV, [^18^F]FDG uptake (kBq/ml)/(injected dose [kBq]/patient weight [g])). BAT activity of each region was determined as average SUV (SUVmean) and as SUVtotal (SUVmean times the volume of the region). Dynamic PET data were used to construct time activity curves of the supraclavicular BAT regions, and the aortic arch was used as an image-derived input function. BAT glucose uptake rates were calculated using Patlak curve fitting^28^ and a lumped constant of 1.14^29^.

### Blood analysis

All samples were frozen and stored at −70 °C until analysis. Circulating FGF21 was analyzed using sandwich ELISA (R&D Systems, Minneapolis, MN, USA), and verified using an in-house ELISA as described previously^3^.

### Statistical analysis

Statistical analysis was performed by PASW Statistics version 20.0 for MacBook Pro. Reported data is expressed as means ± SD. Two-tailed paired t-tests were used in order to compare data before and after cold exposure and data before and after intervention. Shapiro-Wilk test was used to assess normal distributions of plasma FGF21 concentrations for both cooling protocols separately. Linear regression analyses were used to identify correlations between FGF21 concentrations and other variables. Backward multivariate linear regression analyses were used to identify significant independent predictors for BAT activity.

### Study approval

The Ethics Committee of Maastricht University Medical Centre+approved the study protocol and all subjects provided written informed consent. Procedures were conducted according to the principles of the Declaration of Helsinki.

## Results

### Subject characteristics

Two different types of individualized acute cooling protocols, water-cooling and air-cooling, were used in our studies. Subject characteristics for the two types of cooling protocols are presented in Table 1. Note that the air-cooling protocol was performed in male subjects only. Body composition was not significantly different between the two groups of males.

The increase in energy expenditure upon acute cold exposure (expressed as % non-shivering thermogenesis [NST]) and BAT activity in male subjects was not different between the cooling protocols, whereas females showed a lower BAT glucose uptake rate than males (p = 0.009; Table 2). It is important to note that the air-cooling protocol was performed in the morning, whereas the water-cooling protocol was performed in the afternoon. Given that FGF21 levels display a strong circadian rhythm^30^,^31^ with decreased FG21 levels during the day, we chose to analyze results for the two protocols separately. Indeed, in male subjects FGF21 levels at thermoneutrality were significantly higher in the air-cooling protocol (performed in the morning) compared to the water cooling protocol (performed in the afternoon): 85.0 ± 57.0 vs. 48.0 ± 24.8 pg/ml, resp., p = 0.008). For both protocols, male FGF21 levels were normally distributed according to Shapiro-Wilk test (2 outliers were excluded in the air-cooling group).

### FGF21 and BAT activity upon acute cold exposure and other interventions

Female subjects trended towards higher circulating FGF21 concentrations compared to males at thermoneutrality (73.4 ± 46.9 vs. 48.0 ± 24.8 pg/ml resp., p = 0.071), the difference becoming statistically significant during cold exposure (79.6 ± 46.0 vs. 37.9 ± 24.0 pg/ml resp., p = 0.007; Table 2). For both males and females in the water cooling group FGF21 concentrations did not change significantly upon acute cold exposure, whereas FGF21 decreased modestly, but significantly in the air-cooled group (males only) upon cold exposure (from 78.0 ± 48.0 to 70.8 ± 44.3 pg/ml, p = 0.006, Table 2).

Given that FGF21 levels display a strong circadian rhythmicity as mentioned above, it is difficult to conclude whether acute cold exposure has an effect on FGF21 levels, as such an effect may be confounded by the circadian decrease in FGF21. Therefore, we determined plasma FGF21 levels in two other studies, in which we investigated the effect of acute interventions other than cold on BAT activity. Specifically, we recently demonstrated that systemic β-adrenergic stimulation by infusion of the nonselective β-agonist isoprenaline^24^ did not activate BAT. In addition, we showed that ingestion of a high-calorie meal (50% of daily required energy intake) increases glucose uptake in BAT, although this was not related to thermogenesis 25. For both experiments, we determined FGF21 levels at baseline and after 2 hours of intervention. We found that FGF21 levels decreased significantly upon both isoprenaline infusion (from 100.0 ± 52.9 pg/ml at baseline to 63.2 ± 32.3 pg/ml at 110 min after start of infusion, p < 0.001; n = 12), and after intake of a high-calorie meal (from 92.2 ± 45.6 pg/ml at baseline to 65.6 ± 33.5 pg/ml at 120 min after meal ingestion, p = 0.003; n = 10, one outlier was excluded from analysis). The decreases in FGF21 levels in these two studies were much more pronounced compared to the decrease in FGF21 levels upon acute cold exposure (from 78.0 ± 48.0 to 70.8 ± 44.3 pg/ml), suggesting that acute cold exposure blunts the circadian decrease in FGF21 that is normally seen in the morning.

### FGF21 concentration relates to BAT activity

We next investigated whether FGF21 levels and BAT activity during acute cold exposure were related. Given that blood samples between the cooling protocols were collected at different time points we analyzed correlations for each protocol separately.

**Figure 1:**
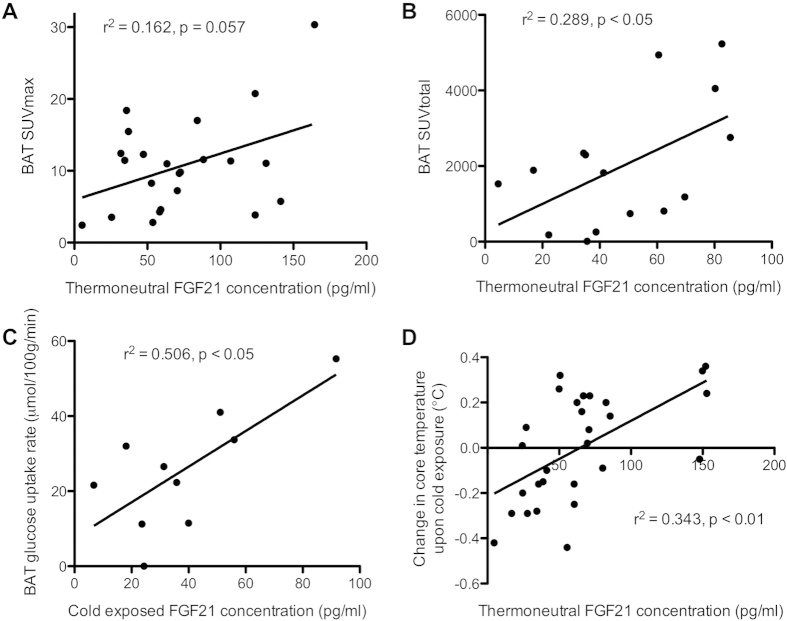
Serum FGF21 concentrations are related to BAT activity. Relationship between basal serum FGF21 concentrations and measures of BAT activity in (**A**) male subjects of the air-cooling group and (**B**) male subjects of the water-cooling group. A positive correlation was also found between FGF21 concentrations during cold exposure and BAT glucose uptake in the male subjects from the water-cooling group (**C**). Basal FGF21 levels correlated positively with the change in core temperature upon cold exposure (**D**).

In the air-cooling protocol (males only), thermoneutral FGF21 levels tended to be correlated with several quantitative measures of BAT activity: SUVmax (r^2^ = 0.162, p = 0.057; Fig. 1A) and SUVmean (fixed volume analysis; r^2^ = 0.222, p = 0.056). Thermoneutral FGF21 levels and BAT volume and activity were significantly correlated in the male subjects of the water-cooling group; BAT volume (r^2^ = 0.278, p = 0.043), SUVtotal (r^2^ = 0.289, p = 0.039; Fig.1B), and a (non-significant) trend was observed with BAT glucose uptake rate (fixed volume analysis; r^2^ = 0.247, p = 0.059). When FGF21 levels during cold exposure were used, the latter correlations became significant; BAT volume (r^2^ = 0.527, p = 0.017), SUVtotal (r^2^ = 0.596, p = 0.009) and glucose uptake rate (fixed volume analysis; r^2^ = 0.506, p = 0.021; Fig. 1C).

In addition, multivariate linear regression analysis in these males, including thermoneutral FGF21 levels, age, BMI and fat percentage as independent variables, showed that FGF21 level was the only significant independent predictor for total BAT volume (p = 0.043) and activity (SUVtotal; p = 0.039).

Interestingly, when including the female subjects in the water-cooling group, the relationships between FGF21 and BAT activity were no longer significant. Moreover, when data from female subjects alone (n = 17) were considered, no associations between FGF21 concentrations and BAT activity were found, indicating a sex specific relationship. In none of the individual cohorts nor in the entire population were any relationships between FGF21 levels and cold-induced NST observed.

Interestingly, basal FGF21 levels correlated positively with the change in core temperature upon cold exposure for both males (r^2^ = 0.49, p = 0.008) and females (r^2^ = 0.27, p = 0.049) in the water-cooling group (r^2^ = 0.343, p = 0.001 for both sexes together; Fig. 1D), and a similar trend was observed in the air-cooling group (r^2^ = 0.159, p = 0.059). Thus, high levels of FGF21 are associated with an increase in core temperature, while low levels of FGF21 are associated with a decrease in core temperature upon cold exposure.

### Increased FGF21 and BAT activity after cold acclimation

**Figure 2:**
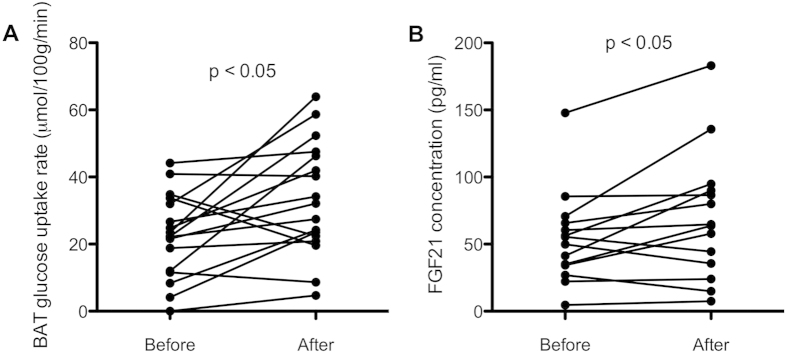
BAT activity and FGF21 concentrations are increased after a 10-day cold acclimation period. BAT glucose uptake rate (**A**) and basal serum FGF21 concentration (**B**) are increased after a 10-day cold acclimation period.

We have recently shown that 10 days of cold acclimation leads to an increase in BAT activity in lean, healthy subjects^13^. Here, we determined if FGF21 was also increased by cold acclimation. Indeed, in parallel with increased BAT activity (Fig. 2A), basal FGF21 levels were increased from 54.0 ± 34.4 to 70.2 ± 47.7 pg/ml (p = 0.028; Fig. 2B), while FGF21 levels during cold exposure were also slightly higher after cold acclimation, although the latter did not reach significance (63.0 ± 39.2 vs. 72 ± 39.2 pg/ml, p = 0.23). After cold acclimation, the significant correlation between BAT activity and FGF21 levels in the basal (SUVtotal; r^2^ = 0.806, p = 0.002) and cold exposed (SUVtotal: r^2^ = 0.507, p = 0.048) state in male subjects was still present. However, the change in FGF21 concentration upon 10 days of cold acclimation did not correlate significantly with the change in BAT activity (r^2^ = 0.357, p = 0.156).

## Discussion

Our data show that serum FGF21 levels correlate positively with cold-induced brown adipose tissue activity in humans. Interestingly, this correlation was only present in male subjects. Multivariate analysis revealed that the level of FGF21 is the strongest determinant of maximal BAT activity. In addition, we show that FGF21 levels are increased upon a 10-day cold acclimation period, in parallel with increased BAT activity. These results indicate a strong link between circulating FGF21 levels and BAT activity upon cold exposure, supporting the notion that FGF21 is a cold-induced activator of BAT^23^. Also of interest is the positive association between serum FGF21 levels and the change in core temperature upon cold exposure, indicating a novel role for FGF21 in defending core temperature in the face of reduced ambient temperature, possibly via activation of BAT.

FGF21 has emerged as an important potential target for the treatment of the metabolic complications associated with diabetes and obesity^32^,^33^. Due in part to its inherently complex biological action studies have documented positive effects on glucose and lipid metabolism, including improvements in insulin sensitivity, lower blood lipid and glucose levels and preserved β-cell function in mice^2^,^5^,^7^,^34^ and improved plasma lipid profiles, reduced insulin levels and body weight in humans^10^. Furthermore, FGF21 has recently been shown to be involved in human cold-induced thermogenesis. Lee *et al.*^23^ suggested that this effect is mediated by FGF21-induced activation of BAT. Although they used a very limited number of subjects (n = 5), they also showed that the diurnal reduction in circulating FGF21 levels was blunted upon mild cold exposure and that this blunting effect was markedly greater in BAT-positive compared to BAT-negative subjects^23^. The positive correlation between basal FGF21 levels and BAT activity that we found in our study in a large cohort confirms a role for FGF21 in BAT activation.

The apparent lack of effect of acute cold exposure on plasma FGF21 levels would contradict a role for FGF21 in cold-induced BAT activation. However, it is known that FGF21 levels have a strong circadian rhythm, with a fall in FGF21 levels in the morning. Although we are limited by the lack of a paired control group in which FGF21 levels are determined under thermoneutral conditions without any other intervention, acute cold exposure did blunt the FGF21 diurnal decrease when compared to two other short-term interventions (isoprenaline infusion and high-calorie meal intake), in which FGF21 levels did drop. Although blood drawing time points were slightly different during these intervention and we cannot exclude that isoprenaline infusion and/or meal intake could be responsible for the decrease in FGF21 levels, we feel it is more likely that this decrease is due to diurnal variation in FGF21 levels, as our data nicely fits data that has been published before^18^,^35^. Moreover, it was recently shown that meal intake did not influence the diurnal decrease in FGF21^35^. Therefore, it is most likely that indeed cold exposure is able to blunt the circadian decrease in FGF21, although it remains to be established whether FGF21 has a direct effect on activating BAT in humans. Specifically, we would suggest that human clinical interventions with infusion of recombinant FGF21 are needed to reveal potential direct effects of FGF21 on BAT activity. It has previously been hypothesized that the elevation in energy expenditure following pharmacological FGF21 exposure is responsible for the rapid weight loss observed with this agent given that no effect on food intake is reported with treatment^32^,^36^. It is important to consider that in patients treated with an FGF21 analogue weight loss was also evident^10^, and it will be interesting to determine whether FGF21 mediated elevation of energy expenditure, possibly due to an effect on BAT, is translatable to man.

In addition to the effects of acute cold exposure, we show that a 10-day cold acclimation period leads to elevated plasma FGF21 levels accompanied by increased BAT activity. Therefore, it is tempting to speculate that this prolonged cold exposure is sufficient for BAT to exert effects on systemic FGF21 levels via secretion of FGF21 into the circulation, as has been shown in rodents^19^. On the other hand, we did not find any direct relationship between the changes in FGF21 levels and BAT activity upon cold acclimation. How cold acclimation in humans could increase FGF21 levels is unknown. During the 10-day cold acclimation period, especially at the first few days, subjects often reported shivering, thereby mimicking active muscle contractions during exercise. It has recently been shown that a 2-week exercise intervention also increases serum FGF21 levels^37^, likely due to PI3-kinase/Akt1-mediated FGF21 secretion by skeletal muscle^38^. It is conceivable that shivering can induce similar FGF21 secretory pathways in skeletal muscle that may partly account for the increased serum FGF21 levels after the 10-day cold acclimation period.

In summary, taken as a whole our data show that plasma FGF21 levels in humans are associated with cold-induced BAT activity. We go on to show that cold acclimation increases both serum FGF21 levels and BAT activity. Further studies are needed to reveal a direct effect of FGF21 on BAT activation; however, if successful, therapies aimed at improving metabolic health via induction of BAT thermogenesis should consider FGF21 as an important target.

## Author Contributions

M.J.W.H. performed experiments, analyzed data and wrote the manuscript. E.B. analyzed data and wrote the manuscript. R.J.S., M.J.V., A.V.D.L., A.C.A. and C.C.C. contributed to data acquisition and data analysis. W.D.V.M.L. and P.S. contributed to the study design, interpretation of data and reviewed and edited the manuscript. All authors contributed to the critical revision of the manuscript and approved the final version.

## Additional Information

**How to cite this article**: Hanssen, M. J.W. *et al.* Serum FGF21 levels are associated with brown adipose tissue activity in humans. *Sci. Rep.* **5**, 10275; doi: 10.1038/srep10275 (2015).

## Figures and Tables

**Table 1:** Subject characteristics.

	**Air cooling**	**Water cooling**	
	**Males (n = 25)**	**Males (n = 16)**	**Females (n = 17)**
Age (y)	23.1 ± 3.6	22.3 ± 2.8	22.2 ± 3.5
Body weight (kg)	72.6 ± 7.6	71.1 ± 8.9	61.4 ± 6.9^A^
BMI (kg/m^2^)	21.9 ± 1.8	21.9 ± 2.2	21.1 ± 1.6
FFM (kg)	59.8 ± 5.8	60.3 ± 7.9	43.3 ± 3.7^A^
Fat mass (%)	16.3 ± 4.0	14.7 ± 2.9	28.1 ± 4.9^A^
BMR (kcal/day)	1706 ± 140	1784 ± 164	1459 ± 127^A^

^A^p < 0.01, males vs. females within the water-cooling group.

**Table 2:** Serum FGF21 concentrations, BAT activity and energy expenditure under thermoneutral conditions and mild cold exposure.

	**Air cooling**	**Water cooling**	
	**Males (n = 25)**	**Males (n = 16)**	**Females (n = 17)**
FGF21 (pg/ml)			
Thermoneutral	78.0 ± 48.0	48.0 ± 24.8^B^,#	73.4 ± 46.9#
Mild cold	70.8 ± 44.3^A^,*	37.9 ± 24.0^B^,**	79.6 ± 46.0^C^
*Static [*^*18*^*F]FDG-PET analysis*			
[^18^F]FDG uptake in BAT (SUVmean)	2.4 ± 0.4	2.6 ± 0.4	2.2 ± 0.9
[^18^F]FDG uptake in BAT (SUVmax)	11.3 ± 6.8	14.2 ± 6.7	10.7 ± 6.8
*Dynamic [*^*18*^*F]FDG-PET analysis*			
Glucose uptake rate in BAT (μmol/min/100 g)	N/A	7.9 ± 3.0l	5.1 ± 2.8^C^
*Fixed volume analysis*			
[^18^F]FDG uptake in BAT (SUVmean)	7.4 ± 5.4	9.0 ± 4.6	6.7 ± 4.2
Glucose uptake rate in BAT (μmol/min/100 g)	N/A	25.4 ± 14.9	14.8 ± 9.8^C^
Energy expenditure (kJ/min)			
Thermoneutral	5.0 ± 0.4	5.2 ± 0.5#	4.2 ± 0.4^C^
Mild cold	5.5 ± 0.5^A^	5.8 ± 0.5^A^,#	4.6 ± 0.6^A^,^C^
NST (%)	11.7 ± 7.3	13.1 ± 8.9#	9.5 ± 8.0

^A^p < 0.05, mild cold vs. thermoneutral;

^B^p < 0.05, air cooling vs. water cooling;

^C^p < 0.05, males vs. females within the water cooling group.

^#^n = 15,

^*^n = 20,

^**^n = 10. N/A, not measured in these subjects.
